# Digital mental health strategies used by young people in Aotearoa New Zealand during the COVID-19 pandemic: ‘Just do it yourself, DIY’

**DOI:** 10.1177/20552076241260116

**Published:** 2024-07-25

**Authors:** Kerry Gibson, Susanna Trnka, Monique Jonas, Pikihuia Pomare, Shauney Thompson, Jemaima Tiatia-Siau, KDee Aimiti Ma'ia'i, Miriama Aoake, Thibaut Bouttier-Esprit, Imogen Spray, Sanchita Vyas

**Affiliations:** 1 School of Psychology, University of Auckland, Auckland, New Zealand; 2 Social Anthropology, University of Auckland, Auckland, New Zealand; 3 Population Health, University of Auckland, Auckland, New Zealand; 4 Psychology, 6420 Massey University, Auckland, New Zealand; 5 Psychology, University of Auckland, Auckland, New Zealand; 6 Māori and Pacific Studies, The University of Auckland, Auckland, New Zealand; 7 Pacific Studies, University of Auckland, Auckland, New Zealand; 8 Campus Life, University of Auckland, Auckland, New Zealand

**Keywords:** Adolescents, digital, social media, mental health, apps

## Abstract

**Objective:**

With rising rates of mental health distress amongst youth during the COVID-19 pandemic, digital resources have been identified as a valuable tools for delivering support to young people. However, many of the websites and apps developed by professionals to support the youth do not take account of the importance young people place on exercising their own agency in managing their mental health. This article investigates how young people in Aotearoa New Zealand used digital resources to manage their mental health needs during the COVID-19 pandemic.

**Methods:**

The study gathered information from semi-structured interviews with 34 young people aged 16–22 years. The data was analysed using reflexive thematic analysis.

**Results:**

Six themes were identified including: searching for online information about mental health; evaluating digital mental health resources; controlling mood through online activity; looking for escape in the virtual world; staying connected online; and giving and receiving support.

**Conclusion:**

Young people's practices demonstrated their investment in their own agency, a general reluctance to engage with professional resources and recognition of the need to balance the risks and benefits of the informal strategies they preferred. Young people appeared sceptical of professionally-designed mental health resources and interventions and preferred to adapt and re-purpose the wide range of platforms and networks available in their informal digital worlds.

## Introduction

Mental health professionals and researchers have identified the potential to reach young people in distress via digital resources and have focussed attention on designing web or app-based psychoeducational resources and interventions designed to support youth mental health. However, there is still much to learn about whether, and how, young people use digital resources to manage their mental health. During the COVID-19 pandemic, with young people experiencing increased stress and reduced access to other forms of support, digital strategies for dealing with mental health took on particular importance.^1–3^ This article explores the strategies that young people used to manage their mental health during the COVID-19 pandemic in Aotearoa New Zealand (NZ), highlighting their own agency in the ways they engaged with the wide variety of digital resources available to them.

### Young people, mental health, and agency

There is widespread international concern about rising rates of mental health problems amongst youth,^4^ usually defined as being between the ages of 15 and 24.^5^ However, despite young people having one of the highest rates of mental health problems, estimates suggest that less than 30% of those experiencing a mental health problem will make use of professional support.^6,7^ Young people have long been recognised to rely on their own resources or informal peer or community support rather than engaging with a mental health professional.^8^ Their reluctance to use formal mental health services has been attributed to a range of factors including practical impediments to access, concerns about negative judgement, and mental health services that are poorly designed for this age group.^9–11^ In this body of research, however, one of the most cited reasons given for young people's poor use of mental health services is their investment in their own autonomy and a preference for self-reliance.

Developmental theories identify the struggle to achieve autonomy as central to adolescence,^12^ but it has been argued that this preoccupation now extends into early adulthood as young people spend more time in education and confront a range of economic barriers to independence.^13^ While young people's quest for autonomy is often represented as a universal developmental phenomenon, it can be more usefully understood as a response to social conditions. In Western countries like NZ, young people are given mixed messages about their autonomy. While discourses associated with neoliberalism suggest that young people have considerable choice about how they live their lives, this is in marked contrast to many young people's experiences of being subject to the authority of their parents, teachers, university lecturers, and employers, as well as broader social and economic constraints that limit their choices.^14^

The lack of power that young people experience in their everyday lives is echoed in their engagements with mental health services.^14,15^ There is a long tradition in these services of excluding people with mental health problems from decisions about their own mental health.^16^ Young people are seen as a vulnerable and risky population and are particularly likely to be positioned as recipients of care rather than being recognised as active participants in managing their own mental health.^17^

Resisting passive representations of people with mental health problems, some theorists have drawn attention to agency as an important ingredient in people's engagement with professional knowledge and resources.^18,19^ We prefer the use of the word agency rather than the narrow developmental notion of autonomy in relation to young people, as it validates the importance for all people, including young people, for being able to act on their own conscious choices. While the concept of autonomy is often treated as a personal capacity, agency recognises the way that people's ability to act is located in particular sets of power relationships.^20^ There are, however, complexities in the use of agency to describe young people's behaviour. Deterministic accounts of youth often ignore agency and position young people as helpless victims of society, and their own intentions as irrelevant to mental health outcomes.^21^ In contrast, humanistic notions of agency tend to overstate young people's free will and their abilities to make choices outside of social and economic constraints.^17^ It is helpful perhaps to step outside of this dichotomy, and to think of young people as being able to exercise contextual agency^22^ or, what has also been called, socially situated agency.^23^ These concepts recognise that young people are influenced by broad social forces but are, nonetheless, actively engaged in negotiating the day-to-day situations in which they find themselves. Through their agency, young people are able to contest the operation of power as well as appropriate and use available resources in ways that match their priorities.^23^ This conceptualisation of agency paves the way to explore young people as reflexive actors who actively negotiate their mental health needs.

### Youth mental health and the digital world

Young people's high usage of the internet has drawn considerable attention from clinicians and researchers with an interest in youth mental health.^24^ The research in this area reflects the contrasting views on youth agency in relation to their mental health. Many researchers and mental health clinicians have focused on concerns about the negative impact that social media and gaming have on young people's mental health.^25–27^ In this view, young people are often depicted as subject to the addictive power of gaming or social media, enacted through algorithms designed by nefarious commercial interests. Researchers also highlight a range of other risks including the potential to be exposed to bullying, grooming and sexualisation, social comparison, and unhelpful ideas about mental health in online social networks.^28–30^ While many of these concerns are valid, they might underestimate young people's ability to recognise and negotiate the risks of the digital environment in which they have grown up.^14^

Ironically, even while researchers and clinicians have raised concerns about the negative impact that digital activity might have on young people's mental health, there has been a rush to exploit the potential of these new technologies to reach young people in distress.^31^ Researchers have identified distinct advantages in apps and e-therapies, recognising the capacity of this new medium to overcome the barriers that prevent young people from using real-world mental health services, drawing attention to the accessibility of these resources, the potential to minimise stigma, as well as the opportunity for young people to exercise greater control over their engagement with mental health resources in the digital environment.^14,32,33^

There is a burgeoning body of research evaluating professionally-designed mental health apps intended for youth populations, but these might not always be in touch with young people's digital practices and preferences. Evaluations, for example, tend to focus on young people's engagement with a single platform or intervention offering mental health support.^34^ However, research on young people's engagement with the internet suggests that they seldom engage with a single digital platform and rather treat the digital environment like a buffet from which they can select and curate their own resources.^35^ Furthermore, the focus of research on young people's engagement with professional mental health resources often ignores their preference for informal, non-professional resources and interactions.^14,36^ This suggests that young people are likely to be engaging with a much broader array of resources than professionals suppose. Finally, evaluations tend to assume that young people will engage with professional mental health resources in the way that the designers intended. This has been challenged by recent research which suggests that young people de-centre professional views in their use of digital resources and focus on their own priorities in selecting and re-purposing these to meet their needs.^37^ Research also indicates that young people's engagement with the wide array of resources enabled by the internet, is shaped by their own identity, their search for authentic connection, the importance of trusting relationships, as well as an investment in making their own choices.^14^

Researchers have argued that it is precisely the opportunity to act outside of the influence of adults who typically assert control within their lives which makes digital activity so attractive to young people.^38^ From the perspective of mental health professionals, the internet is often seen as a ‘wild West’ with potential risks to mental health.^39^^(p328)^ However, from the perspective of young people, digital spaces allow them greater freedom to explore their mental health and to find options that they feel are a better fit for their own needs.^14^ It is vital to understand more about what digital mental health resources young people are actually using and how they negotiate the potentials and risks of this unregulated terrain.

### The context of COVID-19 in NZ

NZ had one of the most effective responses to COVID-19 but this entailed some of the strictest and longest lockdowns of any Western country.^40^ Between 2020 and 2022, schools, universities and many places of work were intermittently forced to close and operate remotely where possible. During this period young people had much of their schooling and university study online, conducted their friendships almost completely virtually, and sometimes engaged in part-time paid work remotely too. Many young people found themselves at home for long periods of time, with only family or flatmates for company, or in school or university dormitories with a limited range of social interaction. A recent report on the impact of COVID-19 on schools in NZ suggests that this period was stressful for young people who noted fears about illness as well as the challenge of dealing with the demands of schoolwork under these conditions.^41^ Other reports noted young people struggling with academic demands, financial pressures, isolation, and tense or toxic home environments during the pandemic.^41–44^ Young people were reported to be particularly vulnerable to the mental health effects of the pandemic, with a large NZ survey conducted in 2020 noting higher rates of distress for those between the ages of 18 and 24 than for other age groups.^45^ However, while rates of distress increased, fewer people felt able to use the mental health services available, especially in Auckland where the effects of the pandemic were most pronounced.^46^

Without access to professional support, many people were forced to rely on their own resources to manage the mental health impacts during this challenging period.^47^ Given their familiarity with online spaces, young people reportedly turned to digital resources to help them manage their mental health.^1^ While the pandemic created unusual circumstances, it also provided a unique opportunity to understand how young people might be using digital resources to help them through the challenges of this time. The current study was planned prior to the pandemic with the aim of understanding young people's digital mental health practices, but with data collection coinciding with the most significant health and social impacts of COVID-19 in NZ (between 2020 and 2022), we took the opportunity to explore how this context was shaping young people's practices. This article addresses the question of how young people used digital resources to manage their mental health during the pandemic, with a particular focus on how they enacted their agency and negotiated the challenges and opportunities in this virtual world.

## Methodology

Our research was underpinned by a social constructionist epistemology which recognises that mental health and practices to support well-being are defined differently depending on the time, context, and social group. While we recognised that there were many ‘youth cultures’ that might influence young people's digital practices differently, for this article we wanted to focus on some of the commonalities we identified across our diverse sample of young people. Our approach to the research was also influenced by a youth-empowerment orientation which prioritises the importance of hearing young people's voices in matters that concern them.^48^ This position entailed a reflexive awareness of our own positioning as mental health professionals and researchers. The younger members of the research team conducted interviews to minimise the power-imbalances that commonly affect the relationship between researchers and youth participants. Their age, which was closer to that of the participants and their familiarity with the digital worlds of young people, afforded the advantages of an ‘insider’ perspective on the research topic. The senior research team included researchers who were of Māori and Pacific Island decent to ensure cultural safety for indigenous participants and interviewers were culturally matched to these participants.

### Research design

The data that form the basis of this article were collected as part of a broader study on young people's digital mental health practices entitled: ‘Ka Hao te Rangatahi: Fishing with a New Net? Rethinking Responsibility for Youth Mental Health in the Digital Age’. This NZ-based study aimed to explore a diverse range of young people's experiences of using digital resources to manage their mental health, as well as the perspectives of mental health app developers and policy makers. The research was approved by the University of Auckland Human Participants Ethics Committee in 2020 for 3 years. Particular ethical considerations in this study included the mental well-being of the participants which was overseen by the two clinical psychologists on the team. The clinical psychologists provided guidance on the ethics protocols to ensure the psychological safety of participants, they trained interviewers to respond to sensitive mental health issues and were available to provide advice and a referral to a professional if a participant appeared distressed and in need of further support. All participants were provided with a list of mental health resources they could access at the conclusion of the interview. A further priority was the confidentiality of young participants who, under pandemic restrictions, were often obliged to participate in research interviews in their environments which allowed little privacy. All participants gave written informed consent for their involvement in the research. As all participants were over the age of 16, ethical guidelines did not require parent or guardian consent.

### Recruitment

Purposive sampling was used in an attempt to ensure that the cultural diversity of NZ youth was well represented. Information on the research was provided to youth organisations (schools, youth groups, and university societies) in Auckland, Northland, Wellington, Nelson and Christchurch who were asked to distribute advertising material to their members via email, notice boards, or social media. The advert for participation was also circulated via social media through networks that the younger researchers on the project were part of (e.g., Facebook groups for young Pasifika or Māori people, Reddit networks for those interested in gaming). Adverts called for people between the ages of 16 and 22 years to take part in an interview to discuss how young people were using digital resources to deal with their mental health. Potential participants were asked to contact the researcher on a dedicated research phone. Those who expressed an interest in taking part in the research were emailed a participant information sheet that provided details about the researchers and the aims of the research.

### Participants

There were a number of interviews with young people collected during the broader study, but for the purposes of this article we have included only the 34 interviews conducted during the period of the pandemic. Participants included 15 young women, 16 young men, and three participants who identified as non-binary. The age range of participants was 16–22 years. All participants were based in one of NZ's major cities, but some were identified as having come from rural areas to study at a city university. All participants were studying either at a school or university, and many worked part-time. Six participants were Māori, 12 had Pacific Island heritage, eight participants identified themselves as NZ European, and the remaining participants came from a variety of migrant backgrounds including India, China, Vietnam, Latin America, and South Africa. While researchers often refer to saturation- a concept associated with Grounded Theory- to justify their sample size, Malterud's notion of ‘information power’ was better suited to our study. Information power is a function of the breadth of the study aim, specificity of the sample, utilisation of an established theory, dialogue quality and analytic strategy.^49^ Given the breadth of our study aim and relative nonspecificity of our intended sample, application of theory (youth empowerment), high-quality dialogue and cross-case analytic strategy, a sample size with the mid 30s was judged sufficient to yield rich data.

### Data collection

We provided participants with the option of engaging in interviews through a variety of mediums including in-person interviews, video interviews, and digital messaging interviews. We had allowed for these different mediums in the light of previous research suggesting that while some young people were comfortable discussing the sensitive issue of mental health in-person, others, especially those who spent much of their time online, felt more able to discuss sensitive topics via text.^50^ Video interviews were intended to address practical barriers to accessibility. The digital messaging option also provided greater protection for the privacy of those who did not have the option of a private space in their homes to conduct a remote video. As it transpired, under COVID-19 restrictions, the majority of our participants either chose or were obliged to conduct their interviews using video (Zoom) or instant messenger (WhatsApp). Interviews were collected intermittently between July 2020 and November 2021. At times recruitment was challenging as the impact of the pandemic was experienced by different communities.

The interviews were designed with the participation of all members of the research team, with particular attention to the suggestions of younger researchers. The interviews were piloted prior to use and revised to ensure they were youth-friendly and culturally appropriate for diverse groups of participants. The interviews covered key content areas including basic demographic information, content, websites, or apps participants used to manage their mental health, and how they used these. We also asked questions about how participants understood mental health and how their circumstances, including those related to COVID-19, affected their practices. The semi-structured format of the interviews allowed each interviewer to adopt a flexible format, allowing for the exploration of areas of interest as they emerged.

The interviews varied in length. The verbal interviews were between 30 and 90 minutes long with most of them being between 50 and 60 minutes. The instant messaging interviews tended to be about 90 minutes long, with a range between 35 and 150 minutes, although one continued for a few days as the participant struggled with an erratic internet connection.

### Data analysis

Our approach to data analysis drew from Braun and Clarke's reflexive thematic analytic approach which is well-suited to identify common patterns in qualitative data.^51,52^ Verbal interviews were audio recorded and transcribed in full. The verbal interviews were largely transcribed by the interviewer but a small number were transcribed by a professional service and checked by the interviewer. The instant messenger interviews were automatically transcribed using WhatsApp technology. Careful reading and rereading of the data enabled us to identify codes in the data and then combine these into themes as recommended by Braun and Clarke.^51^ The analysis was conducted manually, without the aid of software. We applied a reflexive approach to data analysis, in which we explored the youth perspectives present in our data in relation to our research questions and theoretical framework informed by a youth empowerment perspective and recognition of youth agency. The researcher reflexivity emphasised in Braun and Clarke's more recent work allowed us the opportunity to reflect critically on the ways in which our own positioning influenced our understanding of the data,^52^ and we were able to share ideas and refine the analysis through consensual discussion.^53^ We benefited from ‘insider’ insights into the worlds of young people shared by our younger team members.

## Findings

The analysis generated six themes that captured the variety of ways that participants used digital resources to manage their mental health during COVID-19.

### Searching for information about mental health problems: ‘I think I know myself’

Most of the participants acknowledged that they had been drawn to looking up mental health information online during the pandemic to try and make sense of their distress but, on the whole, seemed sceptical about the value of professionally designed resources and information. Many participants acknowledged that they had searched for mental health information online to see whether they might meet the criteria for a diagnosis. Despite this, the majority were clearly cautious about accepting what ‘Dr Google’ might say. As one participant put it:

And, I think everyone knows that you don’t look up symptoms online and trust what you find anyway. But, sometimes I’ll be curious and look it up anyway because it might give me some idea of what's happening.

Although others expressed interest in seeing whether their experience fitted the diagnostic criteria, they emphasised their own agency in determining or accepting a diagnosis. One young woman, for example, gave an account of how she turned to a professionally developed app to find out whether she really did have ‘depression’ as she suspected she might:

I have this other app on my phone that's kind of… you enter in your symptoms and then you answer a series of questions. It asks about how often this happens, and then it kind of calculates what condition you’re dealing with.

However, she quickly clarified her intention to establish what she felt she already knew about herself rather than seeking a professional opinion:

I mean, I think I know myself enough to know what kind of mental issues I deal with. I did at first use that app to test if it would come out with that result. And it did, so I was like, ‘oh wow, okay, this is cool.’

Other participants were also curious to explore what online resources could tell them about their mental health but expressed a similar degree of caution about the authority of professional advice they found on the internet. One participant, for example, conveyed his view that mental health advice should not necessarily be regarded as reliable or applicable to everyone:

If I’m anxious and I’m not sure what to do I’ll google search remedies or healthy habits to help with my anxiety. Sometimes it works but most times it's only best for that person and they can only suggest it to others.

In contrast to their scepticism about the information they found on mental health websites, participants seemed more convinced by accounts from people who had experienced mental health problems themselves, as one participant explained:

I used to watch YouTube videos of people who did have those illnesses and try to compare and contrast their symptoms and experiences with mine. It was helpful only because I found a lot of similarities.

Participants seemed aware that these sources did not necessarily have professional standing but, nonetheless, experienced these as relatable and comforting. Part of the appeal of these informal resources was that they reassured participants that they were not alone with their mental health struggles:

I have watched YouTube videos before to hear about other people's experiences just to know that I wasn’t alone with what I was experiencing.

For some participants it was particularly important to hear about mental health problems from people who shared an identity with them. One participant, for example, spoke about how they preferred to hear about mental health from people who looked like them, and lamented the lack of ‘brown faces’ talking about these issues online:

I think if there were more brown people who were open to … kinda talk about mental health and their troubles… I think it would influence a lot of other… more brown people to feel comfortable to open up to people.

While participants spoke about mental health information that corresponded with traditional psychiatric labelling, others appeared to define mental health more broadly, searching for knowledge that would help them make sense of their distress in different frameworks. One participant, for example, explained how they found it most helpful to make meaning of their distress from a philosophical perspective:

YouTube is used to look into stuff like philosophy and individuals who share knowledge I find valuable. Granted though the videos I watch don’t touch on mental health specifically but they help me understand why someone would feel a certain way about things, and that helps me understand myself.

### Evaluating digital mental health resources: ‘a foreign thing’

Participants described actively looking online for ways to cope with their mental health during the pandemic. However, while most were aware that there were a range of professionally designed online resources and apps specifically designed to help people deal with their mental health, very few participants seemed to be using these. As one participant summarised:

I think there are more tools available to us. But as to whether or not we’re utilising them, I don’t think we are um, utilising them as much as the people who have developed them want us to.

One frequently cited reason for participants’ reluctance to use professional mental health resources was that they were seen as being driven by commercial interests and participants said that they did not want to pay for this kind of support. One participant described how they had used a well-known mental health app but had stopped because of the cost:

I don’t really like paying for apps because that's just, like, an ongoing expense and I don’t work during uni because I don’t have the time for that. I like to focus on my studies so I don’t want to, like, be paying for an app that maybe I won’t get the most use out of.

Besides the cost, however, it seemed that participants also perceived these professional apps and tools as intruding into their own digital exploration of their mental health. They conveyed concerns that professional resources might challenge their independence and bring with them the threat of external professional involvement:

I wouldn’t look online. Haven’t looked at any apps or anything. Um, I don’t know. I think there's the scary prospect of it is telling someone you don’t know about it.

This same participant went on to suggest that apps would be better if they were designed by people who had mental health problems rather than by professionals whom he clearly associated with other forms of unwanted authority in his life:

It might kind of help to be made by people that have issues with mental health, so it doesn’t just seem like a foreign, you know, like a foreign thing or something that you’d see at school.

In addition to these apps being seen as an external invasion of their virtual worlds, some participants seemed uncomfortable about the demands that apps made from them. One young person, for example, described how she had stopped using an app that required her to log her water consumption, something she believed was important for her mental health:

I don’t want to be thinking about having to go into an app every single time I do something.

Besides these deterrents, participants also referred to a stigma associated with using apps set up as being about ‘mental health’, something they did not experience in more informal digital interactions:

I imagine like, if I had a mental health app on my phone or something, I would be quite like, almost like kind of anxious about it being on there and people seeing it.

A few participants described using meditation apps or resources but many of their accounts suggested some discomfort in this experience. One young man, for example, conveyed his embarrassment about having found a meditation app useful:

A friend was like, ‘Oh I’ve got this meditation app, you should try it’. And I tried it and they do these, this is going to sound really lame…listening to someone's monotone voice talk about like you know, the scenery and breathing exercises. It does all these things. But I didn’t realise how much it kind of helps.

An emphasis on agency was visible amongst the few other examples where participants had used a meditation app. One participant, for example, explained how important it was for her to be able to choose voices she preferred on the app she used. Another participant dismissed the idea that the use of a meditation app could be part of a strategy to manage her mental health, insisting that she only used it to help her ‘get to sleep’.

One participant captured the ambivalence that many of the participants seemed to feel about relying on professionally designed resources to manage their mental health, emphasising his preference for informal strategies that fitted his needs:

And I think there's a lot of people out there that are similar to me… just do it yourself, DIY, you know?

### Controlling mood through digital activity: ‘I can watch something funny’

Rather than relying on professional mental health resources, participants seemed to prefer repurposing their existing digital activities and platforms to consciously manipulate their mood. One young person, for example, described how they selected a very particular content because they were familiar with the effect it had on them:

I can always guarantee that if I watch an episode of this show or this anime, I'll feel better walking out on it… because I was able to guarantee a form of happiness.

Participants seemed highly aware of the range of distressing emotions they were trying to manage through their choice of digital activity. The young man quoted above described watching an episode of anime as an antidote to the loneliness he felt during lockdown:

So, it's kind of a substitute, you don't need to feel like you're alone if you just get into that sort of thing and I guess that's what really helped me, like, especially during the lockdown. Like I stay in my room a very long time.

Another spoke about how they used gaming to deal with feelings of anxiety that arose during the pandemic:

I’ve started developing quite a big anxiety, like this year. And I know that I was, and I tried to use video games as a distraction for that.

Many participants referenced music as something that could be relied on to shift their mood state, using platforms like Spotify and YouTube to select their musical choices. One young man, for example, explained how music helped him to deal with the challenge of isolation during the pandemic:

When I start becoming anxious and I think of my mental health as well, ‘cause I’ve, in the past few years, you know, I’ve just been in my room alone. And it's just like, it's a bit, you know, with music it just helps me to bring it a bit up, you know, keep going forward from that situation.

While mostly participants talked about the way they used digital resources to change their mood state, several discussed selected music to match or enhance a particular mood, as though this allowed them to more easily express how they were feeling. One participant explained how they had ‘an upbeat party playlist’ for when they were feeling good, and ‘a sad buzz playlist’ to reflect their mood when they felt down.

Although participants often turned to digital resources to manage their mood, they also recognised a need to do this through ‘real-life’ activities. One young woman described how in the earlier COVID-19 lockdowns she had tended to spend most of her time online, but in later periods of lockdowns she realised that this did not always work for her:

So like if I start seeing the signs of like me slipping back into my old bad habits, I can be like, I need to do something about this, you know I need to like get out and walk around or something just keep myself awake and alive.

Participants also spoke about how they had become more aware of how their physical health affected their mental health and the importance of taking steps outside of the virtual world to maintain their overall wellbeing:

I realised that I wasn’t really keeping up with my [fitness routine], like, I gained weight throughout the first lockdown. But then after the second lockdown, I realised that I need to focus on my physical wellbeing, especially to help with my overall mental health.

Like these participants, others were also clearly monitoring the helpfulness of using digital resources to manage their mood, weighing up the benefits and risks of this as the following example from a participant suggests:

I just go for a walk, yeah. That's during exam time because I kind of just don’t wanna tune into video games at that time, yeah. ‘Cause then my brain will start thinking about stuff to do in there rather than just studying.

Participants also appeared to be aware of the potential for online material to create a negative mood and avoided sites and interactions that had the potential to do this. Several participants spoke about how they had left social media platforms or networks to avoid some of the negative feelings they had while engaging with them. One young man made it clear that he would stop playing a video game if it made him feel unhappy:

If a game made me feel bad I wouldn't play it, I guess. I would just be like ‘Oh, I got bored of this.’

### Looking for escape in the virtual world: ‘somewhere else that's different to everyday life’

Participants also chose to distract themselves from the stresses they faced over the pandemic period using a variety of digital resources. After a day of trying to keep up with digital learning and assignment schedules in the midst of COVID-19, ‘mindless’ online recreational activity provided some relief. One young man, for example, described how he used gaming as a conscious way of escaping his own expectations of himself:

I was getting so stressed about my uni work that I was, like, you know what, I just need a break ‘cause I’ve been overworking myself way too much. So like, I played last night for a few hours with my friends. And, it just made all my worries go away in those few hours.

One of the advantages of these virtual activities seemed to be that they enabled participants to imagine a world different from the one they were living in. One participant, for example, spoke about how he and his friends sought out games that did not mimic the challenges of the real world, poignantly imagining a world in which money was not an issue:

I know it's like, almost like an escape from the, the reality, that they’re like you know, their everyday lives basically. A lot of people would use that as an escape you know… to an island where you don’t have to worry about money.

In contrast to the heavy burden of responsibility that young people felt for maintaining their academic performance or earning an income, their digital ‘escape’ strategies were valued precisely because they held little social or economic consequence.

Some participants also spoke about using digital technology to escape the boredom of lockdowns where there was little to occupy them. One participant, for example, explained how gaming helped to fill the gap that had been previously filled by his studies and friends:

So I gamed a lot more to fill in that space during lockdown…so I didn’t really have anything to do. I couldn’t work so it was just games were a good way to fill in that space.

Participants appeared to make a conscious choice to engage in essentially meaningless activity that did not have effects on their life, as the following account of gaming suggests:

It's not going to have any impact, as in if I could spend three hours doing it and I could do brilliantly or I could spend five hours doing it and do terribly. So that's not going to impact anything else in the rest of my life. …‘cause there's no consequence essentially.

However, a number of participants who spoke about the advantages of ‘switching off’ with digital technology, also recognised the potential for this to become a ‘bad habit’. One young woman who had spoken about how ‘mindless scrolling’ through her social media accounts allowed her to disengage from pressure, explained that she recognised that this was not always helpful:

I have read a lot of kind of wellbeing Instagram posts that say distraction can be helpful when you’re dealing with [a problem] when you’re in that kind of mindset. But the way I see it, distraction for me is actually unhealthy because I end up distracting myself and then it's not addressed.

Despite recognising the downsides of distracting themselves from their problems in this way, participants conveyed a sense that, on balance, this was a necessary and understandable way of escaping the pressures of their lives during the COVID-19 pandemic.

Too much of it is really bad for your mental health but I feel like for lots of young people that can be something that they can go do and get their mind off stuff and you know just be in their like happy place I guess.

### Staying connected online: ‘it's difficult to meet them in real life’

Social connection was seen as central to participants’ mental health. As many opportunities for social interaction were disrupted or limited altogether during the pandemic, participants explained that they turned to digital connections to sustain friendships. One young man, for example, acknowledged that he had substituted social connections in-person, with social activity via gaming:

…lockdown does impact it more to be [honest], I rely on gaming for the social activity. Like if I'm actually going to school and meeting new people whatever I don't feel the need to sit down and have a session of [a game] with friends.

Another young person spoke about how keeping connected to friends on social media helped them deal with the anxiety and uncertainty of the pandemic, and provided reassurance that the people they cared about were okay:

So I did definitely use it a bit over lockdown just to see what updates were, to see things like that. See how family is going over in England and things like that. So yeah I definitely used it more over lockdown than I would necessarily.

During lockdown periods, many young people were also trapped inside their homes with their families for extended periods of time and digital social interaction was a way of temporarily escaping this environment as one young man explained:

I guess it was good to hear someone else's voice that isn't your parents, or your siblings…I think being cooped up inside is not something that is the most ideal for people.

Participants spoke about how they were able to create a sense of community across the range of digital mediums available to them, including text messaging, gaming with others, watching videos together, and sharing screens as they talked, as one participant explained:

So, as well as voice chatting there are also, like, text channels but you can post pictures. And, we have channels where my friends just post pictures of the games like, not so for work or for news. Or like funny things or memes. And, it feels like a, yeah, it feels like a real community. It's quite nice.

Participants also appeared to derive comfort from just knowing their friends were ‘there’, without the need for active communication. One participant, for example, described how he and his friends felt less alone when they live-streamed their screens to one another as they engaged in different activities:

So that we can see what each other is doing. Like, my friends are making music or I’m just looking at some worky things on the internet.

Despite enjoying the sense of connection they found online, participants also seemed aware of the way that polarised and toxic communication had escalated during the pandemic, and valued the option that social media provided to ‘mute or block or report’ people they did not want to interact with. One young woman gave this articulate explanation of how she differentiated between safe and unsafe communities when she sought a connection:

I think probably much in the same way that physical spaces do, it gives a lot of [choice]. If you curate your experience right you can sort of have a feed or a blog or a group of friends or whatever, that does see you as you are or has the same interests, and that is socially affirming. And then also you have more mainstream social media that are more mainstream society I guess, like you see your crazy aunt like, ‘vaccines are here to control us,’ and you’re like, ‘no, Aunt Linda, please stop’.

Interestingly, this example suggests that participants recognised that real-world communities (including relatives) might have the potential to be toxic, rather than this simply being a product of the digital world.

But as much as participants were able to curate and control their digital activities, some also spoke of the loss of being able to spend time in-person with their friends as they might have in the past, as one young man wistfully acknowledged:

It has made me, like, more eager to see my friends in real life. I wouldn’t say I’m significantly sadder because, I guess, I’m quite used to, like, just working online anyway. But I do miss, like, seeing my friends’ faces, even if we do have some Zoom calls.

### Giving and receiving support in digital networks: ‘I’ll be here for you’

Although just ‘hanging out’ online seemed a common strategy for coping with the mental health challenges of the pandemic, participants also described how online interactions offered the opportunity for valuable reciprocal support during this difficult time. As one participant put it:

But, then there's also things where it's connecting you to other like-minded people that can support you when you need it and people that you can support when they need it. That's been really good. Yeah being able to feel like there are other people going through the shit that you’re going through.

Some participants noted that there seemed to have been an increase in the extent to which people were supporting one another in digital networks, using these to ‘to check in with people and make sure that they’re okay’. One young woman described how she had noted other people reaching out to her in this period in a way that they had not previously done:

Something that I’ve noticed a lot this past eight, ten months is that that's definitely switched around. And I could leave my phone and I will start getting people wondering how I’m doing and saying they hadn’t heard from me, you know.

While participants had clearly used digital networks to give and receive support prior to the pandemic, the pandemic seemed to legitimise this kind of interaction. Participants spoke about the value of being able to ‘vent’ to others, releasing some of the emotional pressure they felt. Others acknowledged how they had had become more proficient in supporting other people online. One participant described how she had previously been unsure about what to say when someone was struggling with anxiety and depression, explaining that by observing interactions she had become better at doing this:

So I would have things to say like, “I’ll be here for you,” or “you can talk to me about it.” That kind of thing. Yeah. “It's going to be okay.” Lines that would fit that kind of situation.

Participants described how voicing concern more generally for people's mental health had become much more prevalent in their social networks during the pandemic, as the following participant suggested:

I did notice recently people posting a bit more on mental health like kind of reminders, things as simple as wishing people they have a good day. And there were social media accounts I noticed people would post to take care of yourself which I thought was quite cool.

Another participant similarly described how people in her gaming network had tried to be especially kind to one another during this period:

Sometimes when you have a rough day at uni, or you haven’t, like, you haven’t talked to anyone all day ’cause of lockdown. Booting up a game, winning a game and just celebrating with your friends and complimenting each and saying, nice, good job, like, it feels pretty good.

But although opening up about mental health distress to people online was seen to be easier than talking in-person, participants seemed highly aware of the potential for breaches of trust and privacy in online environments. Most participants were clear that the reciprocal support only happened in networks of a few selected people, within which they felt safe. One young woman, for example, described how she filtered her friend groups to ensure that these conversations happened only with people she knew well:

We are quite a close-knit group because we’ve known each other for years…Sometimes some of us just want to get it out there and have us kinda react or respond to it to show our support.

Participants also made it clear that they ensured that sensitive conversations remained private, mostly preferring to communicate through direct messaging rather than in public forums, as one participant explained:

I would never respond publicly. I feel like it would be a bit intrusive and some people may think I’m doing it just for show if they see I’m trying to help on a public post. I would private message them.

Participants also acknowledged the risks of getting caught up in online networks that might be unhelpful for their mental health. One participant described how he had come to see how an online group discussing mental health problems was not helpful for him:

I thought I was going on there to get better, but actually – and sometimes it becomes quite echo chamber-y, because you’re all kind of sharing all of this stuff, … And, you’re not really getting help, per se. You’re just kind of getting people reinforcing those ideas.

While many of the participants spoke about using online support in their informal networks, a number of them expressed the view that this could not quite make up for in-person support, as one participant put it:

I prefer talking to people in-person about mental health issues, especially if they’re serious. Just because then I’d be able to fully express my emotions or vice versa with whoever I’m talking to. And that way if there needs to be a hug or a cry or whatever it is we can do it together in-person.

Another participant reiterated this view, noting that young people in his circles had become disillusioned with the reliance on virtual connection and were instead looking for meaningful, in-person relationships in which they could find comfort and support:

It can be hard to develop a relationship and to feel like someone genuinely cares about you if it's through a device… It's a weird thing… but like our generation is going through this process of like, kind of, rejecting technology. [It's] trying to connect us? But we’re kind of like, “Oh, you know, is it?”

## Discussion

Our study suggests that while young people used digital resources to manage their mental health during the COVID-19 pandemic, they did this in ways that carefully protected their own agency. They expressed reluctance to engage with professional mental health resources available on websites and through apps, treating these with a degree of scepticism. Other researchers have recognised that the association of apps with consumerism makes these less available and desirable for young people with limited economic resources.^54^ However, the cost of apps might be only one part of young people's reluctance to engage with professionally designed mental health resources. Our analysis suggests that economic barriers might be exacerbated by a sense that professional resources represent unwanted, external impingements into their digital worlds. While the young people in this study often used terminology suggestive of a mental health agenda, they appeared to have little intention of subjecting themselves to professional judgements or recommendations, at least in their online worlds. Instead, some of their accounts suggested a concern that professional mental health resources would somehow undermine their own choice, subject them to external control, and potential stigma. These concerns have a strong similarity with the concerns that young people have about engaging with professional services in the non-virtual world.^9,10^

The analysis also suggested that young people might understand mental health more broadly than professionals and are open to exploring a range of resources that they feel support their physical, spiritual, and relational well-being rather than those focused exclusively on diagnosable mental health problems. Awareness of the multi-dimensional nature of wellness might be particularly important for the Māori and Pasifika youth and other similar indigenous communities who hold a more holistic understanding of mental health.^55–57^

Our study also highlights young people's preferences for finding their own ‘DIY (do it yourself)’ strategies for managing the challenges they struggled with during the pandemic. They found their own solutions by re-purposing existing social networks and platforms to meet their needs. They were drawn to activities such as gaming, listening to music, and scrolling through social media content to facilitate the experience of positive emotions and distract themselves from the stresses of the pandemic, including academic pressure, loneliness, feeling trapped at home, and financial challenges. Interestingly, these strategies are not dissimilar to recommendations within professional mental health protocols for distress tolerance, commonly used in Dialectical Behaviour Therapy.^58^ These clinically recommended strategies include recognition of the value of taking time out from stress through distraction, and actively seeking soothing experiences, including music, as a way of managing overwhelming feelings of distress.

The importance that young people place on finding support in their own networks is also consistent with professional recommendations about social support, long recognised as an important antidote to stress and an important protective factor for young people's mental health.^59,60^ The findings of our study suggest that young people were acutely aware of the importance of a sense of connection and support from others during this difficult period. They actively sought opportunities to be with one another online to counteract the enforced isolation of the pandemic. They also made themselves available to receive support from others and to give this when needed. This ethos was consistent with the public health messaging on COVID-19 which emphasised kindness and mutual support.^61^

Importantly, our analysis challenges the common perception that young people will be irresponsible and naive in their unregulated engagement with digital resources.^62^ The young people who took part in this study seemed well aware of the risks of using digital resources to address their mental health and took steps to minimise these. Rather than just accepting information, they appeared thoughtful in their judgements about whether a particular resource would be useful to them. They were conscious of the risks of toxic or unhelpful online interactions, and cautious about the potential to get drawn into digital activities that distracted them from more direct ways of addressing their mental health.

Interestingly, while the pandemic threw the value of digital communication into relief for many adults who had not relied on this previously, for young people it seemed to make them more aware of the constraints of having to depend only on virtual connection or activity and appeared to reignite a longing for the ‘real world.’

The findings of this study have important implications for mental health professionals who are hoping to overcome the barriers to engaging young people with support by using digital resources. While it appears that digital spaces do allow young people the opportunity to exercise their agency, this does not appear to make them more inclined to engage with professionally designed digital resources. As has been found in the real world, young people still prefer to retain control over their own experiences of mental health and their choices about how to manage this.^14^ While, from a professional standpoint, this might be seen as a disadvantage of digital support, this study highlights the care and thoughtfulness that young people put into negotiating their use of the wide array of digital resources available to them, and their awareness of the risks and benefits of these.

Recognition that young people are active agents in seeking, selecting, and using digital resources is an important step in understanding how best their mental health can be supported through this medium. Their concern to protect their own agency does not negate the value of professional knowledge on mental health, but it does suggest a different role for professionals if they want to facilitate young people's finding meaningful support online. Rather than positioning youth as recipients of psychoeducation websites and intervention apps, professionals might work together with young people to develop guidelines for the use of the informal resources they already use and to increase the safety of their interactions with these. Instead of designing professional interventions that might be seldom used, professionals would do better to develop young people's capacity to engage with their digital world in ways that are supportive of their mental health and minimise the risks of this environment. This could, for example, include helping young people become more proficient in evaluating the quality of mental health information available on the internet, choosing what material they engage with, carefully curating their online feeds, or monitoring the time they spend on social media. Strategies could also include building young people's skills to engage supportively with their online social networks. There are already some good examples of how this kind of approach can be implemented. For instance, #chatsafe worked with young people to develop guidelines for the discussion of suicide in their social media networks.^63^ Working closely with young people, it might be possible to match mental health support better to the digital priorities and practices of this population.

## Limitations

While the sample captures a diverse range of NZ youth perspectives, the constraints of this article did not allow for an in-depth analysis of differences within the group. This is particularly relevant for the Māori and Pasifika youth who have higher rates of mental health problems arising due to the impact of colonisation and ongoing systemic racism.^64^ There has also not been an opportunity to explore gender differences, particularly recognising that young men and women might use digital resources differently and the LGBTQ+ community might have particular mental health needs as a result of the discrimination they face.^65^ The specificity of experience within these important groups of young people will be addressed in future articles. Finally, it needs to be acknowledged that while all the data was collected during the two years in which the COVID-19 pandemic was most evident in NZ, there were periods in which both the virus and association restrictions varied in intensity and these effects were also not uniformly felt around the country. The data have largely not been reported with reference to this changing context, except where participants specifically mentioned this.

## Conclusions

This study highlights young people's agency in using digital resources for managing their mental health and their preference for using a wide variety of informal resources rather than relying on professional expertise. While professionals are concerned about young people engaging with mental health in unregulated digital spaces, young people's informal strategies for managing their mental health were often consistent with professional recommendations and the analysis suggested that they actively negotiated and tried to avoid some of the risks of the online environment. There is potential to use digital resources to reach young people in distress, but this needs to be done together with young people and in ways that fit with their own practices, including a respectful recognition of their agency.

## Supplemental Material

sj-docx-1-dhj-10.1177_20552076241260116 - Supplemental material for Digital mental health strategies used by young people in Aotearoa New Zealand during the COVID-19 pandemic: ‘Just do it yourself, DIY’ Supplemental material, sj-docx-1-dhj-10.1177_20552076241260116 for Digital mental health strategies used by young people in Aotearoa New Zealand during the COVID-19 pandemic: ‘Just do it yourself, DIY’ by Kerry Gibson, Susanna Trnka, Monique Jonas, Pikihuia Pomare, Shauney Thompson, Jemaima Tiatia-Siau, KDee Aimiti Ma'ia'i, Miriama Aoake, Thibaut Bouttier-Esprit, Imogen Spray and Sanchita Vyas in DIGITAL HEALTH

sj-docx-2-dhj-10.1177_20552076241260116 - Supplemental material for Digital mental health strategies used by young people in Aotearoa New Zealand during the COVID-19 pandemic: ‘Just do it yourself, DIY’ Supplemental material, sj-docx-2-dhj-10.1177_20552076241260116 for Digital mental health strategies used by young people in Aotearoa New Zealand during the COVID-19 pandemic: ‘Just do it yourself, DIY’ by Kerry Gibson, Susanna Trnka, Monique Jonas, Pikihuia Pomare, Shauney Thompson, Jemaima Tiatia-Siau, KDee Aimiti Ma'ia'i, Miriama Aoake, Thibaut Bouttier-Esprit, Imogen Spray and Sanchita Vyas in DIGITAL HEALTH
