# Supplementary material for: Digital mental health strategies used by young people in Aotearoa New Zealand during the COVID-19 pandemic: ‘Just do it yourself, DIY’
Source: Digit Health. 2024 Jul 25;10:20552076241260116. doi: 10.1177/20552076241260116 (PMC11282513; doi:10.1177/20552076241260116)
Supplement: sj-docx-1-dhj-10.1177_20552076241260116 - Supplemental material for Digital mental health strategies used by young people in Aotearoa New Zealand during the COVID-19 pandemic: ‘Just do it yourself, DIY’ [file sj-docx-1-dhj-10.1177_20552076241260116.docx]

| **Section/Topic** | | **Item No** | **Checklist item** | **Comment** | **Repor ted on**  **page No** |
| --- | --- | --- | --- | --- | --- |
|  | **Domain 1: Research team and reﬂexivity** | | | | |
|  | Personal Characteristics | | | | |
| *Interviewer/*  *facilitator* | | 1 | Which author/s conducted the interview or focus group?Interviewer/facilitator | Shauney Thompson (ST) & KDee Aimiti Ma'ia'I (KA) conducted the interviews with Pasifika youth.  Mariama Aoake (MA) conducted the interviews with Māori youth.  Thibaut Bouttier-Esprit (TB), Imogen Spray (IS) and Sanchita Vyas (SV) conducted all the remaining youth interviews. | N/A |
| *Credentials* | | 2 | What were the researcher’s credentials? E.g. PhD, MD | In addition to research/professional credentials, researchers also had particular cultural knowledge appropriate for engaging with indigenous participants and data.  Kerry Gibson (KG) –PhD, Clinical Psychologist  Susanna Trnka (STR) – PhD, Anthropologist  Monique Jonas (MJ) – PhD, Health Ethicist  Pikihuia Pomare (PP) – DClinPsy, Clinical Psychologist, Māori knowledge.  Jemaima Tiatia-Siau (JT) – PhD, Community Health Researcher, Pacific Island knowledge.  ST – BSc Hons, DClinPsy candidate, Pacific Island knowledge  KA – BA, Research Assistant, Pacific Island knowledge.  MA – BA, MA candidate, Research Assistant, Māori knowledge.  TB – BA, MA candidate.  IS – MA, PhD candidate  SV – BA, BA Hons candidate. | N/A |
| *Occupation* | | 3 | What was their occupation at the time of the study? | KG, STR, MJ were employed as academic staff at the University of Auckland.  PP was employed as academic staff at Massey University.  ST, KA, MA, TB, IS, and SV were all postgraduate students and research assistants at the University of Auckland. | N/A |
| *Gender* | | 4 | Was the researcher male or female? | KG, STR, MJ, PP, ST, KA, MA, IS, and SV all identify as female.  TB identifies as male. | N/A |
| *Experience and training* | | 5 | What experience or training did the researcher have? | KG, STR, MJ, and PP all have extensive experience in conducting qualitative research.  ST, KA, MA, TB, IS and SV all received training in conducting interviews for the current research and did these under supervision of the senior researchers on the project. | N/A |
|  | Relationship with participants | | | | |
| *Relationship established* | | 6 | Was a relationship established prior to study commencement? | None of the researchers had close personal relationships with the participants although some were known to the interviewees through their university peer networks and social media groups. This is inevitable when seeking participants who might be part of minority cultural and social groups and is in line with the Te Ara Tika research ethics framework within which research in Aotearoa is anchored. |  |
| *Participant knowledge of the interviewer* | | 7 | What did the participants know about the researcher? e.g. personal goals, reasons for doing the research | The participants were informed about the researchers’ affiliations and the aims of the research via a participant information sheet which was made available to them prior to their consent to participate. The interviewers were trained to establish a relaxed, informal atmosphere in which the interviews could be conducted. As part of this they were encouraged to be open about their own affiliations and interest in the project, including their own knowledge and involvement in digital practices. | 8 |
| *Interviewer characteristics* | | 8 | What characteristics were reported about the interviewer/facilitator? e.g. Bias, assumptions, | Included reflexive commentary on the position of the senior researchers as professionals and older than participants. Recognised the research assistants as ‘insiders’ in terms of youth culture. Included discussion of indigenous cultural knowledge within the team. | 7, 10 |

|  | **Domain 2: study design** | | | | |
| --- | --- | --- | --- | --- | --- |
|  | Theoretical framework | | | | |
| *Methodological orientation and Theory* | | 9 | What methodological orientation was stated to underpin the study? e.g. grounded theory, discourse analysis, ethnography, phenomenology, content analysis | Social constructionism provided the epistemological base. A youth empowerment approach provided an orientation to the research. The methodology was reflexive thematic analysis. | 7, 9 |
|  | Participant selection | | | | |
| *Sampling* | | 10 | How were participants selected? e.g. purposive, convenience, consecutive, snowball | Purposive sampling techniques were used | 8 |
| *Method of approach* | | 11 | How were participants approached? e.g. face-to- face, telephone, mail, email | Participants responded to recruitment adverts. They were asked to text a dedicated research phone to receive more information and were then emailed a copy of a participant information sheet. Interviews were conducted either in person, on video, or via WhatsApp instant messaging. | 8 |
| *Sample size* | | 12 | How many participants were in the study? | 34 participants | 9 |
| *Non- participation* | | 13 | How many people refused to participate or dropped out? Reasons? | No participants refused to participate or dropped out. | N/A |
| *Setting of data collection* | | 14 | Where was the data collected? e.g. home, clinic, workplace | The majority of interviews were conducted online with the participant in their own residence. Face-to-face interviews were conducted in facilities at the University of Auckland or in a private room at a school. | N/A |
| *Presence of non- participants* | | 15 | Was anyone else present besides the participants and researchers? | No | N/A |
| *Description of sample* | | 16 | What are the important characteristics of the sample? e.g. demographic data, date | Participants included 15 young women, 16 young men, and three participants who identified as non-binary. The age range of participants was 16-22 years. All participants were based in one of NZ’s major cities, but some identified as having come from rural areas to study at a city university. All participants were studying either at school or at university, and many worked part-time. Six participants were Māori, 11 had Pacific Island heritage, eight participants identified themselves as NZ European, and the remaining participants came from a variety of migrant backgrounds including India, China, Vietnam, Latin America and South Africa. | 9 |
|  | Data collection | | | | |
| *Interview guide* | | 17 | Were questions, prompts, guides provided by the authors? Was it pilot tested? | The interview schedule was pilot tested, discussed by the research team, and refined prior to the interviews being conducted. The interview guide was semi-structured, listing a number of key areas to be covered but allowing space to follow up on issues raised by participants | N/A |
| *Repeat interviews* | | 18 | Were repeat interviews carried out? If yes, how many? | No | N/A |
| *Audio/visual recording* | | 19 | Did the research use audio or visual recording to collect the data? | Verbal interviews were audio recorded. WhatsApp instant messenger transcript was used to capture the digital interviews. | 10 |
| *Field notes* | | 20 | Were ﬁeld notes made during and/or after the interview or focus group? | Yes, the interviewers wrote brief field notes. | N/A |

| *Duration* | | 21 | What was the duration of the interviews or focus group? | The verbal interviews were on average between 50-60 minutes. WhatsApp interviews took slightly longer, with an average time of 90 minutes. |  |
| --- | --- | --- | --- | --- | --- |
| *Data saturation* | | 22 | Was data saturation discussed? | We have not used the concept of data saturation but have, instead referred to Malterud’s notion of ‘information power’ which captures a judgement on whether the data is sufficiently rich for the intended analysis. The term and its basic components are described within the paper and citation provided. | 9 |
| *Transcripts returned* | | 23 | Were transcripts returned to participants for comment and/or correction? | Participants were given the option of requesting their transcripts for commenting and editing. | N/A |
|  | Domain 3: analysis and ﬁndings  Data analysis | | | | |
| *Number of data coders* | | 24 | How many data coders coded the data? | 2 researchers (KG & ST) did the initial coding of the data but these were considered and discussed by 6 of the researchers to ensure the data was adequately represented and to allow for a variety of perspectives on the analysis. | 10 |
| *Description of the coding tree* | | 25 | Did authors provide a description of the coding tree? | No – there is no coding tree used in reflexive thematic analysis. We developed provisional themes and considered these using consensual discussion within the research team. | 10 |
| *Derivation of themes* | | 26 | Were themes identiﬁed in advance or derived from the data? | Themes were derived from the data – but informed by the theoretical perspective of the researchers. | 10 |
| *Software* | | 27 | What software, if applicable, was used to manage the data? | The analysis was done manually. No software was used in the process. | 10 |
| *Participant checking* | | 28 | Did participants provide feedback on the ﬁndings? | No | N/A |
|  | Reporting | | | | |
| *Quotations presented* | | 29 | Were participant quotations presented to illustrate the themes / ﬁndings? Was each quotation identiﬁed? e.g. participant number | Yes. Quotations were used to illustrate the themes throughout. Quotations were not identified in order to offer a further layer of protection to the participants, especially those representing smaller ethnic groups within NZ. | 11-25 |
| *Data and ﬁndings consistent* | | 30 | Was there consistency between the data presented and the ﬁndings? | Yes – there is consistency between the data presented and the findings. | 11-25 |
| *Clarity of major themes* | | 31 | Were major themes clearly presented in the ﬁndings? | Major themes were clearly identified. | 11-25 |
| *Clarity of minor themes* | | 32 | Is there a description of diverse cases or discussion of minor themes? | Each theme contains the diverse views of participants and recognition of minor themes. | 11-25 |
