# Supplementary material for: Digital mental health strategies used by young people in Aotearoa New Zealand during the COVID-19 pandemic: ‘Just do it yourself, DIY’
Source: Digit Health. 2024 Jul 25;10:20552076241260116. doi: 10.1177/20552076241260116 (PMC11282513; doi:10.1177/20552076241260116)
Supplement: sj-docx-2-dhj-10.1177_20552076241260116 - Supplemental material for Digital mental health strategies used by young people in Aotearoa New Zealand during the COVID-19 pandemic: ‘Just do it yourself, DIY’ [file sj-docx-2-dhj-10.1177_20552076241260116.docx]

Revised Interview Questions – Young People

Ka Hao te Rangatahi: Fishing with a New Net? Rethinking Responsibility for Youth Mental Health in the Digital Age

Basic demographics:

Name:

Gender:

Pronouns:

Ethnicity:

Age:

Year level:

Where are you living? Where are you from?

Well-being; or ‘being well’

1. How do you define mental wellbeing? What does being well look like for you?
2. What does it mean for you to be well? What does your life look like when you feel well?
3. Have you experienced something that enhanced your wellbeing before? How did you respond? (Eg. Going for a walk for mindfulness, incorporating it into daily routine)
4. Have you experienced something that has disrupted your wellbeing before? What did you do? Where did you seek support? Who did you talk to?

Digital Technologies

1. We’re looking at this idea of ‘digital technologies’ which we define very broadly – social media, gaming online, streaming services, apps and so on. How do you define digital technologies?
2. What apps, sites, streaming services do you regularly use? (Nike Run Club, meditation and sleep apps, period trackers, wellbeing journals, food/health/body accounts, social media sites, astrology apps (Co-Star), YouTube (tutorials/ASMR etc), streaming services like Netflix, Spotify, Apple Music (podcasts etc)
3. How much time per day do you spend online? What do you do when you’re there?
4. How do these ‘digital technologies’ fit into your idea of being well? Are they beneficial

or detrimental to your wellbeing?

Being ‘well’ online

1. When you have a question or concern about your wellbeing (sleep, diet, exercise, stress, mindfulness etc.) where do you look for information? Online, your GP, whānau, friends?
2. When you have looked, or used apps/online sites etc. for information relating to wellbeing, how useful was the information? Which apps/sites were most useful for you? Which were least useful?
3. Are there apps/sites that you and your friends use or have recommended to you? What did they/you find useful about them? How often do you/they use them?
4. How concerned do you think people your age are with being well?
5. Any final comments?
